# Energy Expenditure of a Female Tiger in a Human‐Altered Habitat: Insights From Tri‐Axial Accelerometry

**DOI:** 10.1002/ece3.72898

**Published:** 2026-01-05

**Authors:** Zehidul Hussain, Anjali Thapliyal, Luca Börger, Parag Nigam, Bilal Habib

**Affiliations:** ^1^ Department of Environmental Science and Technology University of Maryland College Park Maryland USA; ^2^ Department of Animal Ecology & Conservation Biology Wildlife Institute of India Dehradun India; ^3^ Department of Biosciences Swansea University Swansea UK

**Keywords:** acceleration, activity, behaviour, coexistence, energetics, human‐dominated landscape

## Abstract

Energy expenditure is central to animal ecology and shapes how individuals balance foraging, movement, thermoregulation and risk avoidance. Large carnivores, like tigers ( *Panthera tigris* ), face high energetic demands for territorial defence, hunting, competition and reproduction. These challenges further intensify in human‐modified landscapes, where habitat fragmentation, prey depletion and human disturbance alter movement and behaviour. To quantify these dynamics at a fine scale, we used high‐resolution tri‐axial accelerometry to examine the energetic and behavioural patterns of a sub‐adult female tiger in the human‐dominated Brahmapuri Forest Division of the Eastern Vidarbha Landscape. The individual was fitted with a GPS collar equipped with an 8 Hz accelerometer, and Vectorial Dynamic Body Acceleration (VeDBA) was calculated as a proxy for energy expenditure. Behavioural states were classified using the Daily Diary Multi‐Trace (DDMT) software, which implements a Boolean decision‐tree framework, and validated using camera traps, field observations, and GPS‐based cluster analyses. We further modelled diel and seasonal variation in energy expenditure across life stages (pre‐ and post‐dispersal) using generalised additive models. Four behavioural states were identified: resting, walking, travelling and hunting. Resting dominated the activity budget (~65%), while travelling peaked in the evening (~58%). Energy expenditure (VeDBA) was higher during the post‐dispersal phase (*p* < 0.001), reflecting increased movement likely associated with territory establishment and defence. Seasonal patterns varied by life stage: during pre‐dispersal, winter and summer exhibited bimodal elevated energy expenditure at dawn and dusk, whereas monsoon showed a unimodal activity peaking in the evening, likely influenced by dense vegetation cover and localised resource distribution. Similar bimodal patterns persisted during the post‐dispersal phase, with dawn energy expenditure lower during the monsoon, while dusk activity was similar across seasons. Our findings highlight the behavioural flexibility of a female tiger navigating a human‐altered landscape and demonstrate the utility of accelerometry for quantifying fine‐scale energetics.

## Graphical abstract

This paper presents the fine‐scale analysis of tiger energetics in India using animal‐borne accelerometer. Our findings capture distinctive activity patterns, energy‐intensive behaviours such as hunting and travelling, and shifts in energy expenditure across life stages (pre‐ and post‐dispersal phases). We demonstrate how tigers' behaviour is influenced by ecological features such as time of day and season.

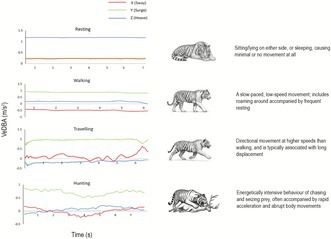

## 1 Introduction

Energy expenditure plays a fundamental role in animal ecology, influencing foraging, hunting, predator avoidance, reproduction and thermoregulation (McHuron et al. 2019; McGrosky and Pontzer 2023). Animals allocate energy across these activities by balancing energetic gain with the costs incurred through movement, risk exposure and physiological demands. As a key component of ecological function, energetic demand shapes behavioural decisions and affects when and where animals acquire resources (Klappstein et al. 2022; Wilson et al. 2022; Nisi et al. 2022). This balance becomes particularly critical for large carnivores, which face high energetic demands due to their large body size, endothermic metabolism and carnivorous diet (Carbone et al. 2011; Forbes et al. 2024; Gavrilov 2024; Ritwika et al. 2024). These demands are further elevated by anthropogenic disturbances in a human‐dominated landscape, which alter the availability of prey, increase movement costs and impose spatiotemporal constraints on behaviour (Thorsen et al. 2022; Hussain et al. 2025). In such a fragmented environment, large carnivores like tigers ( *Panthera tigris* ) experience greater energetic challenges when navigating human‐modified landscapes, where habitat fragmentation, prey depletion and anthropogenic pressures influence foraging and movement patterns (Habib et al. 2021; Hussain et al. 2022; Jhala et al. 2025). Fragmentation also increases the distances travelled to locate suitable foraging or resting areas and reduces habitat connectivity, thereby increasing the metabolic cost of movement (Mills et al. 2023; Gorman et al. 2024). Wide‐ranging species are especially vulnerable to these costs (Tucker et al. 2018), and tigers have been observed to modify their behaviour by travelling farther and faster at night, likely to avoid human disturbances (Habib et al. 2021; Hussain et al. 2022). However, such shifts may help avoid direct human contact but lead to increased energy expenditure and thermoregulatory stress (Walcott et al. 2020; Bryce et al. 2022). Additionally, prey densities in human‐dominated landscapes are often low due to overgrazing, habitat degradation and hunting (Atwood et al. 2011), forcing carnivores to spend more time searching for prey or shift to livestock (Ruiz‐Villar et al. 2023), which increases their risk of conflict with humans (Zimmermann et al. 2005).

In response to these pressures, carnivores frequently alter their behaviour by becoming more nocturnal, avoiding human‐dominated areas or modifying space use (Suraci et al. 2019; Carricondo‐Sanchez et al. 2020; Gorman et al. 2024). These behavioural shifts can lead to increased energy expenditure over time, especially when coupled with human‐induced climate change. Changes in temperature and precipitation patterns can reduce the availability of water and increase the energetic cost of thermoregulation (Walcott et al. 2020). For example, lions ( *Panthera leo* ) have been shown to expand their home ranges under drought conditions in response to resource scarcity (Ferreira and Viljoen 2022). Collectively, these factors create a complex and dynamic energetic landscape that demands behavioural flexibility.

Understanding how large carnivores manage energy expenditure in such environments is crucial for effective conservation and coexistence strategies. Past research has provided insights into tiger ecology, including behaviour, habitat use, prey selection, movement, space‐use patterns and population recovery (Chundawat et al. 2016; Habib et al. 2021; Hussain et al. 2022; Jhala et al. 2021; Karanth 2003; Karanth and Sunquist 2000; Karanth et al. 2004; Sankar et al. 2010; Sarkar et al. 2016; Seidensticker and McDougal 1993). However, these studies offered a limited understanding of tiger energetics, particularly in human‐dominated landscapes. The integration of high‐resolution biologging tools, such as tri‐axial accelerometers, offers new opportunities to fill this gap by linking fine‐scale activity data with energetic costs.

In this study, we used animal‐borne accelerometers to understand the behavioural dynamics and energetic costs of a sub‐adult female tiger in the Eastern Vidarbha Landscape of Central India. In particular, differences in energetic demands between the pre‐dispersal phase (when individuals remain within their natal range) and the post‐dispersal phase (after they establish a territory) are limited. As dispersal involves shifts in space use, movement intensity and risk exposure, it likely involves distinct energetic strategies. Our objectives were to: (1) classify behavioural states and estimate their energetic costs, (2) assess daily and seasonal variation in activity pattern and (3) evaluate differences in energy expenditure between life‐history stages (i.e., pre‐dispersal and post‐dispersal). We hypothesised that activity and energy expenditure would be modulated by diel cycles, seasonal variation and life stage, reflecting flexible behavioural strategies to balance energetic gain with risk avoidance. Specifically, we expected energy expenditure to be highest during the post‐dispersal phase due to movement associated with establishing, maintaining and defending territory, whereas the pre‐dispersal phase would reflect lower energetic demands within the natal range. While this study is based on a single individual, it provides fine‐scale insight into tiger energetics and highlights the potential of accelerometry to inform conservation in shared landscapes. We hypothesised that energy expenditure would be modulated by diel activity, seasonal variation and life stage, reflecting flexible behavioural strategies to balance energetic gain with risk avoidance. Specifically, we expected energy expenditure to be highest during the post‐dispersal phase due to movement associated with establishing, maintaining and defending territory, whereas the pre‐dispersal phase would reflect lower energetic demands within the natal range.

## 2 Materials and Methods

### 2.1 Study Area

**FIGURE 1:**
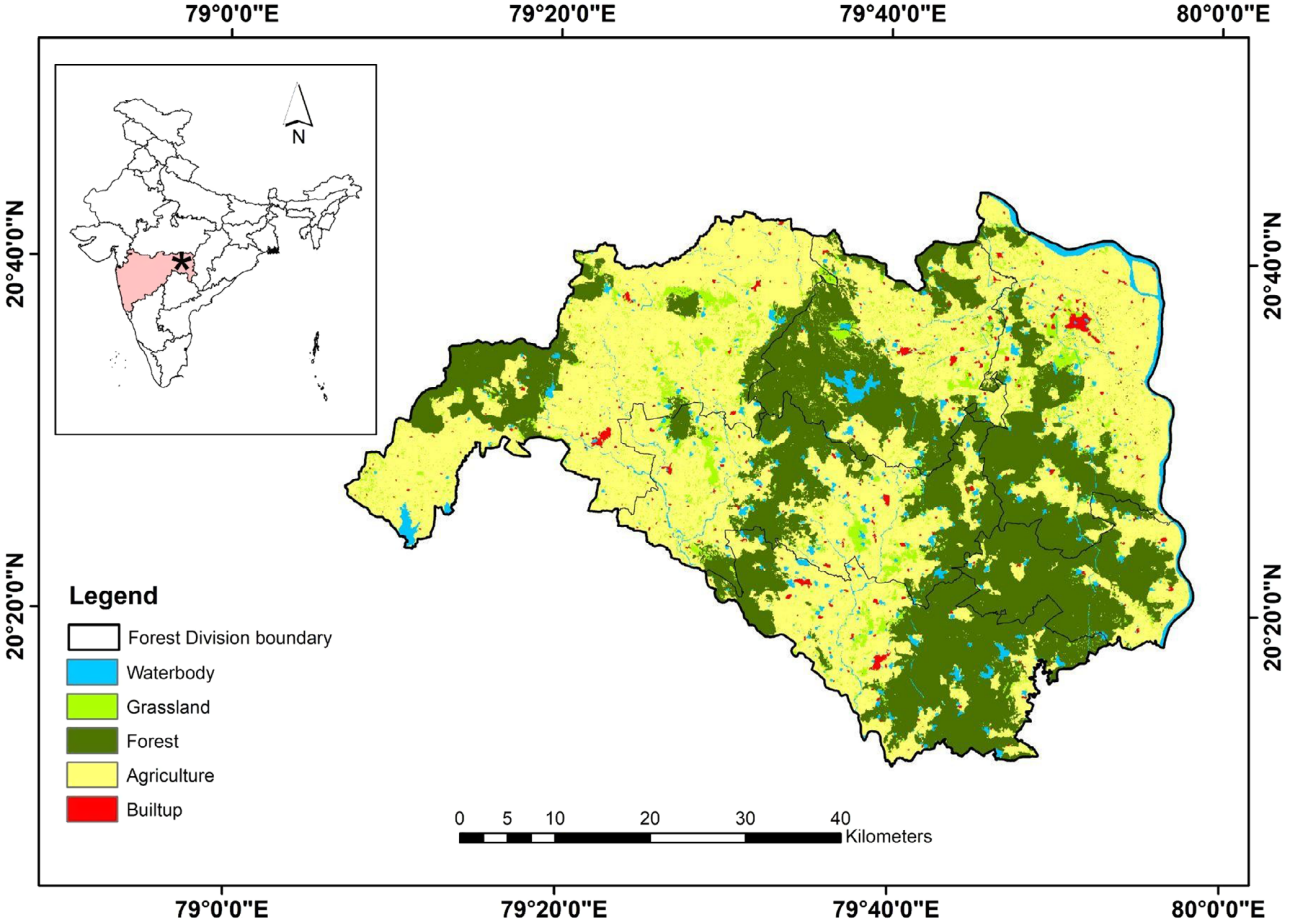
Map showing the study area of Brahmapuri Forest Division, Maharashtra, India. The star on the map of India shows the location of the Brahmapuri Forest Division in the state of Maharashtra.

The study was conducted in the Brahmapuri Forest Division of Eastern Vidarbha Landscape, Maharashtra, part of the Central Indian Landscape (Figure 1). This region spans approximately 1192.87 km^2^ and serves as a critical corridor connecting the Tadoba‐Andhari Tiger Reserve (TATR) in the south to the Navegaon‐Nagzira Tiger Reserve (NNTR) in the east. The area lies outside the protected area (PA) network, and is interspersed with urban and semi‐urban settlements, encompassing approximately 8540 villages (Habib et al. 2017). Despite intense human presence, the landscape supports a viable population of tigers, estimated at 66 ± 1.61 adult individuals, coexisting with other carnivores such as leopards ( *Panthera pardus* ), dholes ( *Cuon alpinus* ) and Indian wolf ( *Canis lupus pallipes* ) along with other prey species like chital ( *Axis axis* ), sambar ( *Rusa unicolor* ), nilgai ( *Boselaphus tragocamelus* ) and wild pig ( *Sus scrofa* ) (Habib et al. 2025). The area is dominated by tropical dry deciduous forest (Champion and Seth 1968) and experiences a tropical climate. Summers (April–June) are typically hot and dry, followed by the monsoon season (July–September), which brings heavy rainfall. Winters (October–March) are cooler and relatively dry (Nandankar et al. 2011).

### 2.2 Capture and Collaring

We captured and collared a sub‐adult female tiger on 28 February 2019, in the Brahmapuri Forest Division in Maharashtra, India. To immobilise the animal, we used a combination of medetomidine hydrochloride, ketamine hydrochloride and xylazine, with dosages estimated from visual assessments of body weight and age. We administered the drugs remotely using an air‐pressurised dart gun (Model IM). The radio‐collaring was part of a more extensive study in which sub‐adult tigers were collared to understand dispersal patterns, space use and movement in a human‐dominated landscape. We fitted the tiger with a GPS‐Iridium collar (GPS Plus, Vectronic Aerospace, Berlin, Germany) programmed to record locations at 1–3 h intervals. The collar included a tri‐axial accelerometer set to record fine‐scale data at a frequency of 8 Hz (eight measures per second), allowing fine‐scale behavioural classification.

### 2.3 Identifying Movements

We classified the sub‐adult tiger's movement into different life stages based on the understanding that dispersal is a three‐stage ecological process, including emigration, transience and settlement (Clobert et al. 2012; Van Dyck and Baguette 2005). We focused on the natal dispersal, defined as the movement from their birth site to the area where an individual eventually establishes a breeding territory (Clobert et al. 2012). We used semi‐variogram plots and the net squared displacement (NSD) to identify the tiger's movement phases (Hussain et al. 2025). The semi‐variogram plots identify range residency by detecting changes in spatial autocorrelation over time, which indicates whether the animal exhibits site fidelity (Calabrese et al. 2016; Fleming and Calabrese 2017). We then applied the NSD method to quantify these changes and classify the movement trajectory into distinct life‐history stages (Börger and Fryxell 2012). Through this combined approach, we identified two key phases: pre‐dispersal, when the tiger remained within her natal range, and post‐dispersal, when she established a stable home range that no longer overlapped with her natal area. Although the data suggested a transitional shift, we did not define a distinct dispersal phase due to the short distance and absence of clear separation between pre‐ and post‐dispersal movement.

### 2.4 Behaviour Classification

We classified the tiger's behaviour using the Boolean time‐based decision tree, known as the LoCoD method, as described by Wilson et al. (2018), and implemented through the Daily Diary Multi‐Trace (DDMT) software (Wildbyte Technologies Ltd., Swansea, UK). The GPS collar tri‐axial sensor recorded acceleration along three perpendicular axes: the *x*‐axis captured anteroposterior (surge or forward/backward) movements, the *y*‐axis recorded dorsoventral (heave or up/down) movements and the *z*‐axis measured transversal (sway or sideways) movements. The collar was deployed for approximately 10 months and retrieved to download the acceleration data. We excluded the first 8 days of data to avoid bias from altered behaviour due to capture stress (Morellet et al. 2009). Using DDMT software, we visualised rhythmic acceleration patterns over time along the three axes and calculated the Vectorial Dynamic Body Acceleration (VeDBA), which serves as a proxy for energy expenditure (Brown et al. 2022). We then formulated Boolean expressions based on the acceleration threshold to classify specific behaviours.

To validate these behavioural classifications, we calibrated the accelerometer data against metadata from the camera trap images, field observations and recordings, and GPS‐based cluster analyses that differentiated resting and hunting events. Based on the GPS movement path, we deployed single‐sided camera traps (*n* = 20) in frequently used areas like trails, near water bodies and dense cover. These time‐stamped images captured behaviours, allowing us to match visual evidence with accelerometer signatures and refine our behavioural categories. We identified clusters of tiger locations using the GPSeqClus package (Clapp et al. 2021), which employs time‐series location data to sequentially aggregate GPS points into clusters based on user‐defined criteria (search radius, temporal window and minimum number of locations). Clusters were categorised as either resting or kill sites (Hussain et al. 2022). We confirmed these kill sites in the field by locating prey remains and other signs of predation, and camera traps were placed at active sites to verify behavioural interpretation. Over the monitoring period, we documented 12 kill sites for the tigress.

## 3 Statistical Analysis

We used VeDBA as a proxy for energy expenditure from the tri‐axial accelerometer data (Grémillet et al. 2018; Qasem et al. 2012). We calculated the dynamic body acceleration (DBA) by subtracting the static acceleration from the raw acceleration values recorded along each axis and summing the dynamic components. We then computed VeDBA as:

$$\text{VeDBA}=\surd \left[{\left(\text{DBAX}\right)}^{2}+{\left(\text{DBAY}\right)}^{2}+{\left(\text{DBAZ}\right)}^{2}\right]$$

We calculated the hourly mean VeDBA across the 24‐h circadian cycle to compare the energy expenditure between the pre‐ and post‐dispersal phases. A non‐parametric Mann–Whitney *U* test was used to assess differences in hourly mean VeDBA between these two phases. To further examine how energy expenditure varied across temporal and seasonal scales across life stages, we modelled hourly mean VeDBA using generalised additive models (GAMs), with time of day (hour) and season (summer, monsoon and winter) as explanatory variables. Time of day was treated as a continuous variable and modelled using thin‐plate regression splines to capture non‐linear diel patterns, while season was included as a categorical factor. We developed five candidate models, each representing a distinct biological hypothesis about how tiger energy expenditure might respond to diel and seasonal factors (Table 1). We fitted all models using restricted maximum likelihood (REML), and the best‐fit model was selected based on the lowest Akaike's Information Criterion (AIC) value. We assessed model adequacy through residual inspection, QQ‐plots and smoothness (k‐index) tests. All statistical analyses were conducted using the *mgcv* package in R version 4.4.1 (R Core Team 2024).

**TABLE 1:** Five candidate generalised additive models (GAMs), each corresponding to a biological hypothesis describing how diel (hour) and seasonal factors (summer, monsoon and winter) shape energy expenditure (hourly mean VeDBA) in a sub‐adult female tiger.

Model name	Formula	Model description
Null	hourlymean_VeDBA ~1	VeDBA is constant; no effect of season or hour
Season‐only	hourlymean_VeDBA ~ season	Season‐only model: VeDBA differs among seasons, but not across hours (no diurnal pattern)
Hour‐only	hourlymean_VeDBA ~ s(hour)	VeDBA varies across the day (diurnal pattern), but does not differ by season
Additive	hourlymean_VeDBA ~ season + s(hour)	VeDBA varies by both season and hour, but the diurnal pattern is the same across seasons
Interaction	hourlymean_VeDBA ~ season + s(hour, by = season)	VeDBA varies by season and hour, and the shape of the diurnal pattern differs between seasons (the hour effect depends on season)

## 4 Results

### 4.1 Behavioural Classification

**FIGURE 2:**
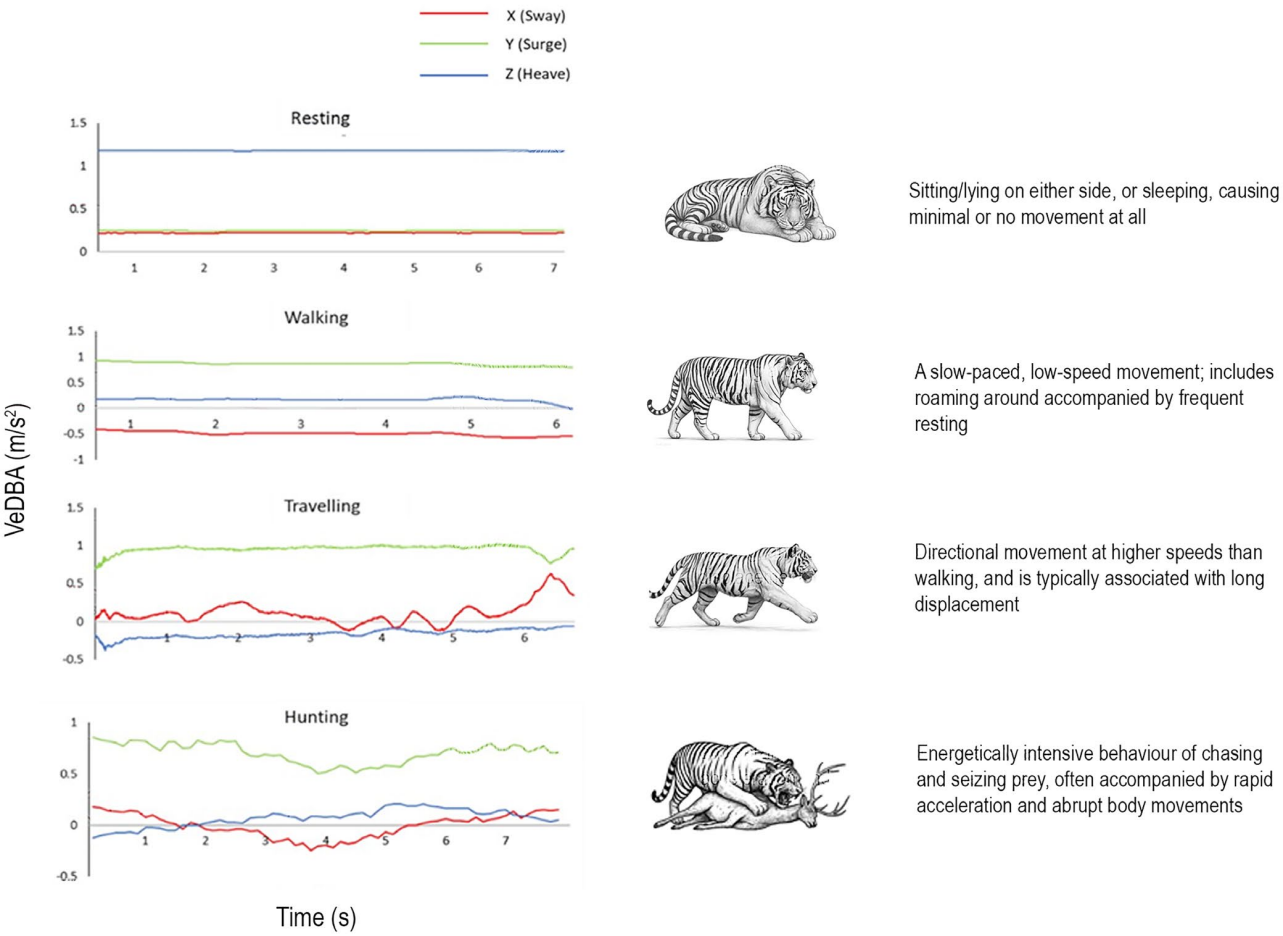
A pictorial illustration depicting the behaviours of a tiger on the right and acceleration bursts on the lateral (red), longitudinal (blue) and vertical (green) axes, for resting, walking, travelling and hunting behaviour.

We identified four distinct behavioural states: resting, walking, travelling and hunting, based on static and dynamic acceleration (Figure 2). Resting behaviour was characterised by stable lying postures for long periods and occasional turns, reflected in abrupt changes in heave acceleration. During resting, VeDBA values remained below +0.001 m/s^2^, indicating negligible acceleration. Sway (*x*‐axis) values exceeded +0.67 m/s^2^, indicating stable lateral movement, while surge (*y*‐axis) and heave (*z*‐axis) values stayed below −0.88 and −0.5 m/s^2^, respectively, exhibiting patterns consistent with low‐movement states. Walking showed increased surge and heave acceleration, with VeDBA ranging between > +0.03 and < +0.06 m/s^2^, while sway values were below −0.4 m/s^2^, reflecting moderate lateral movement. Travelling displayed even higher acceleration, with VeDBA between > +0.1 and < +1.2 m/s^2^, indicating higher energy expenditure compared to walking. Hunting behaviour involved the highest energy expenditure, with VeDBA exceeding +1.2 m/s^2^.

### 4.2 Activity Budget

**FIGURE 3:**
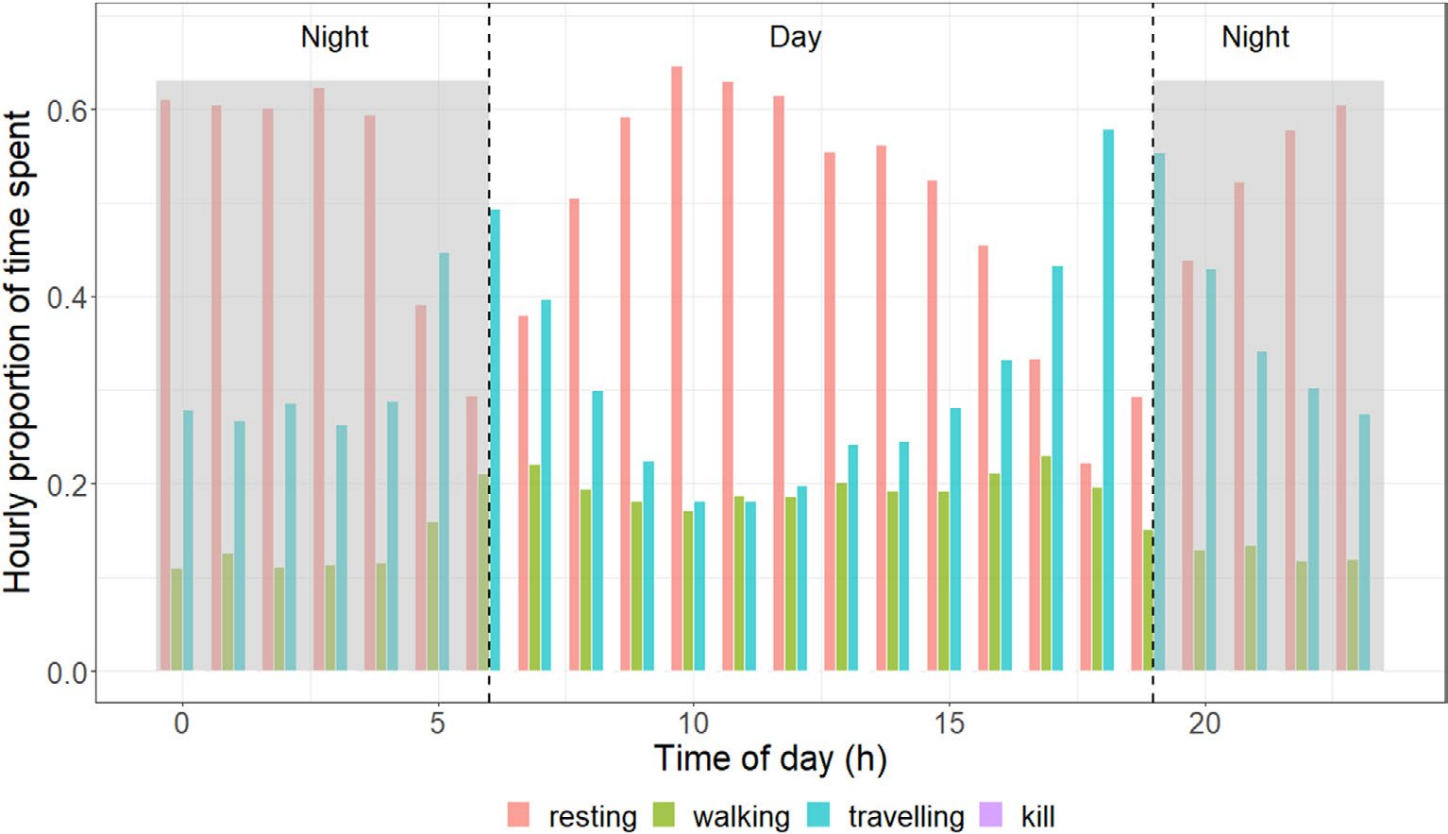
Hourly proportion of activity budget for behaviour classified as resting, walking, and travelling for a sub‐adult female tiger in the Brahmapuri Forest Division, Maharashtra, India.

**FIGURE 4:**
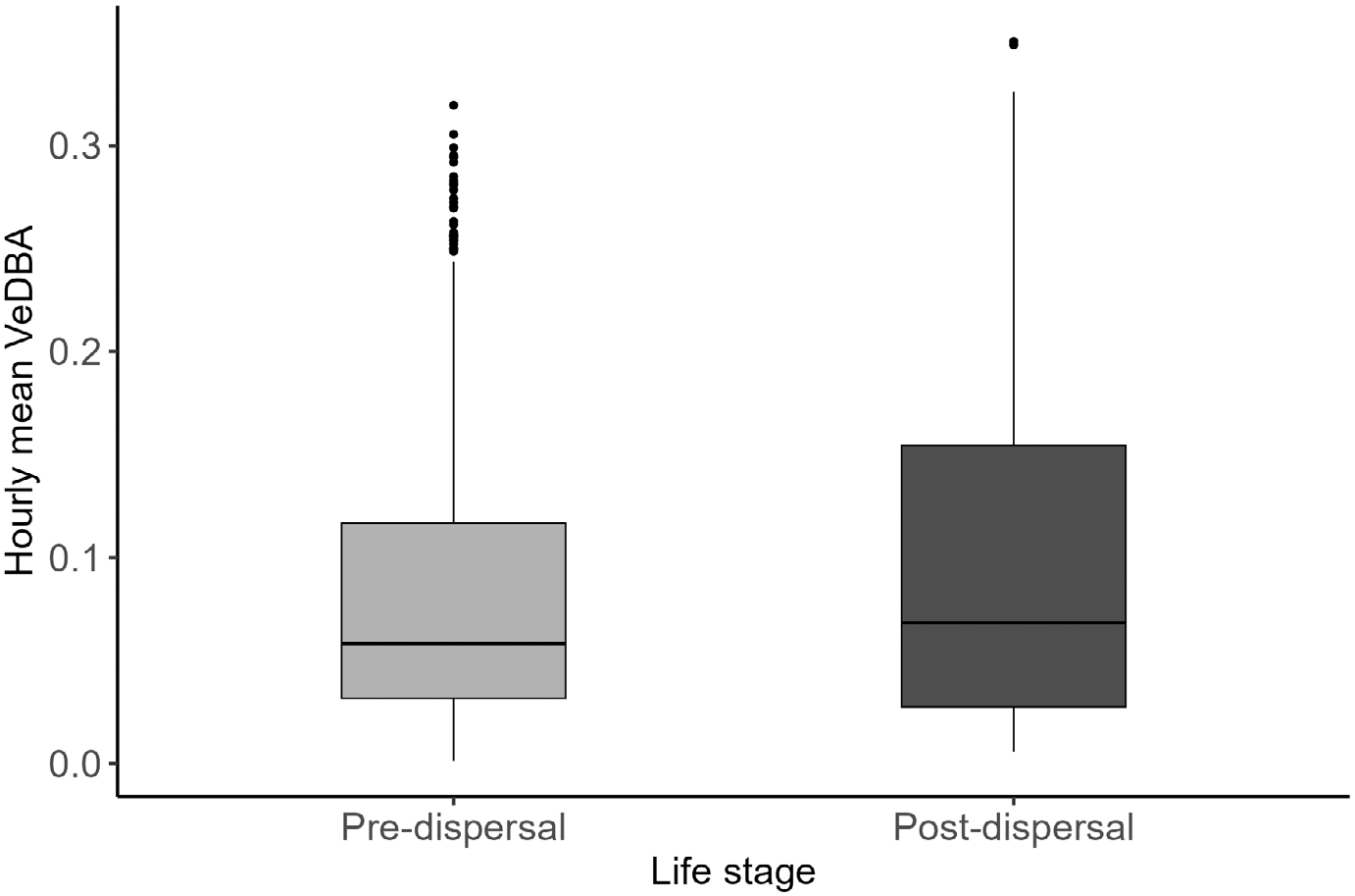
Energy expenditure (hourly mean VeDBA) during the pre‐ and post‐dispersal phase of a sub‐adult female tiger in the Brahmapuri Forest Division, Maharashtra, India.

The overall activity budget showed that the tiger spent approximately 65% of the time resting, primarily during the day (Figure 3). Travelling accounted for about 20% of total activity, with the highest proportion (58%) occurring in the evening. Walking activity peaked at dawn and dusk, while overall movement decreased during midday hours (18%–20%). Hourly VeDBA values were higher during the post‐dispersal phase (median = 0.068 m/s^2^) than during the pre‐dispersal phase (median = 0.058 m/s^2^; Figure 4).

### 4.3 Temporal and Seasonal Variation in Energy Expenditure Across Life Stages

**FIGURE 5:**
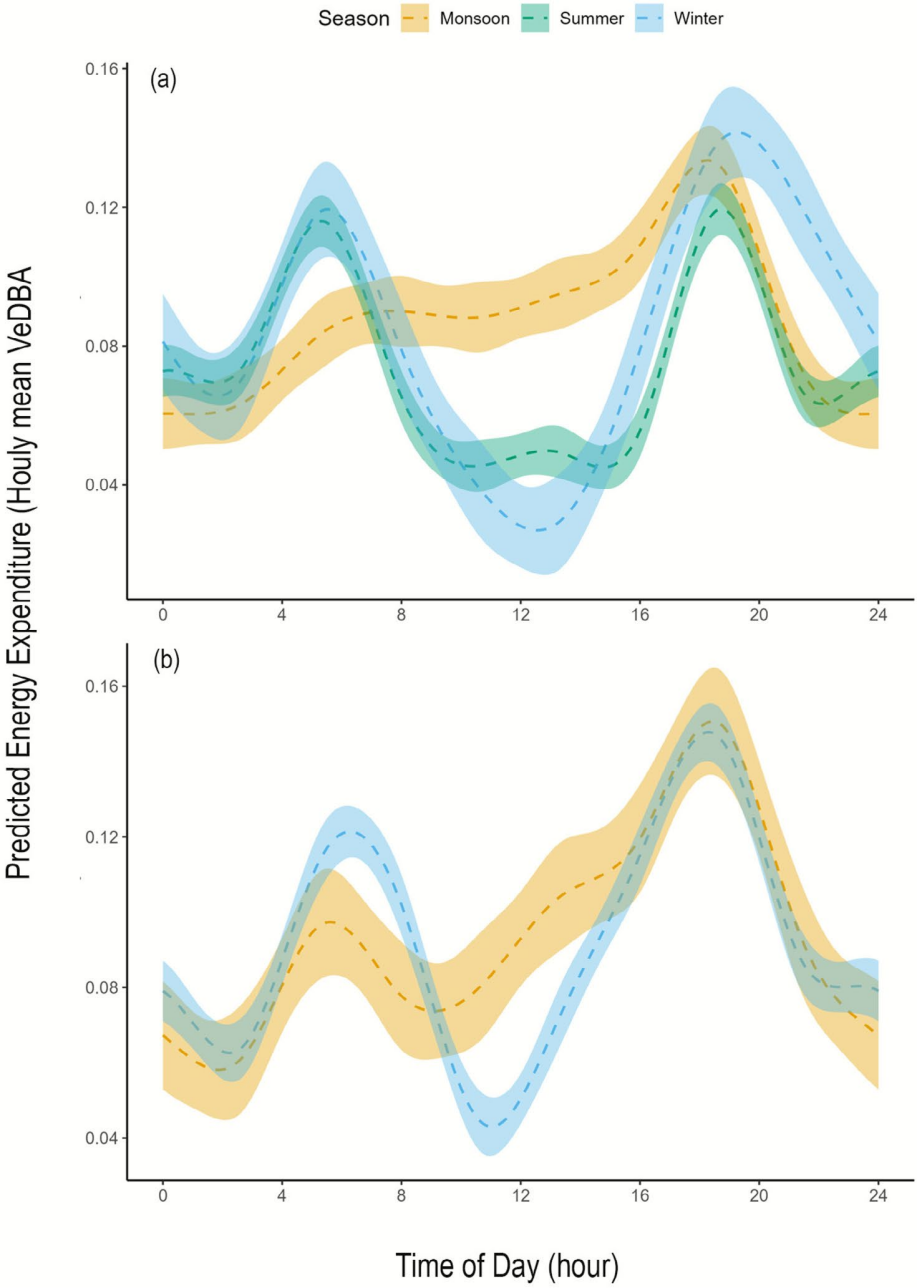
Predicted diel energetic patterns (hourly mean VeDBA) to time of day (hour) for a sub‐adult female tiger across seasons and life stages, based on the best‐supported Generalised Additive Model. Panel (a) shows diel activity curves during the pre‐dispersal period, and panel (b) shows curves during the post‐dispersal period. Lines represent predicted mean VeDBA, and the shaded area shows 95% confidence intervals.

Hourly mean VeDBA was significantly higher in the post‐dispersal phase compared to the pre‐dispersal phase (*p* < 0.001). Similarly, we also found seasonal and diel variation in energy expenditure across both phases, as supported by the best‐fit interaction model. During the pre‐dispersal phase, winter and summer exhibited bimodal activity patterns, with higher energy expenditure at dawn and dusk (Figure 5a). Summer activity was significantly lower than in the monsoon (*β* = −0.0145, *p* < 0.001). In contrast, monsoon activity during pre‐dispersal shifted to a largely unimodal pattern, characterised by elevated activity throughout the day, peaking at dusk between 18:00 and 19:00. Similarly, energy expenditure showed bimodal diel patterns with peaks at dawn and dusk in the post‐dispersal phase (Figure 5b). Energy expenditure during dawn was lower in the monsoon than in winter, while dusk activity remained similar across seasons.

## 5 Discussion

This study provides insights into how a sub‐adult female tiger modulates energy expenditure using tri‐axial accelerometry in a human‐dominated landscape. We examined how behavioural states, diel activity, seasonal conditions and life‐history stage influenced fine‐scale energetic patterns. The predominance of resting (~65% of activity) reflects a clear energy‐conservation strategy, likely adopted to minimise physiological costs associated with movement in a landscape with frequent human presence and habitat fragmentation. Similar behavioural patterns have been documented in other large carnivores, where resting constituted a significant portion of the activity budget, likely to conserve energy while minimising detection risk in disturbed areas (Wang, Nickel, et al. 2015; Wang, Allen, and Wilmers 2015; Suraci et al. 2019).

While resting dominated the activity budget, walking and travelling behaviours followed a bimodal pattern, likely shaped by ecological and anthropogenic factors. Activity peaked at dawn and dusk, periods associated with high prey movement and reduced human activity, offering optimal conditions for foraging in human‐modified landscapes (Karanth and Sunquist 2000; Carter et al. 2012; Suraci et al. 2019). In contrast, the reduction in walking during midday likely reflects a thermoregulatory response, consistent with behaviour observed in other large mammals that minimise movement during peak heat (Owen‐Smith 2008). Walking, which likely facilitated within‐territory movements such as patrolling or foraging, was concentrated during periods of lower thermal and anthropogenic stress, a pattern also observed in large mammals navigating human‐dominated or seasonally dynamic habitats (Dagtekin et al. 2024; Van Cleave et al. 2018). In contrast, travelling exhibited higher VeDBA values, reflecting more intense and energetically costly directional movement peaking during dawn and dusk. Similar high movement rates have been reported for tigers outside PAs, where individuals exhibited higher movement while traversing fragmented, multi‐use landscapes to reduce the time spent in areas with higher human presence (Kertson et al. 2011; Valeix et al. 2012; Habib et al. 2021). These elevated energetic costs are consistent with patterns in other carnivores; for example, pumas show increased energy expenditure during directed movements through non‐forest habitats to reduce exposure to anthropogenic risk (Wang, Nickel, et al. 2015). Furthermore, hunting experienced the highest energetic demand, characterised by short, high‐intensity bursts of acceleration associated with prey capture (Williams et al. 2014; Carbone et al. 2011).

Beyond behavioural state, energy expenditure varied significantly between life‐history stages. During the post‐dispersal phase, the tigress exhibited higher energy expenditure (VeDBA) likely due to increased activity associated with territorial movement and maintaining boundaries against conspecifics. These energetically expensive behaviours are common in wide‐ranging carnivores during dispersal and early territory formation (Mosser and Packer 2009; Behr et al. 2020) (Mosser and Packer 2009; McNutt and Silk 2008; Behr et al. 2020). Nonetheless, across both life stages, energy expenditure followed a bimodal circadian rhythm, with peaks aligning with dawn and dusk, a pattern that balances foraging opportunity with risk avoidance (Karanth and Sunquist 2000; Carter et al. 2012; Van Cleave et al. 2018).

Seasonal and temporal variation in energy expenditure further highlights the behavioural plasticity of the tigress across life stages. During the pre‐dispersal phase, winter and summer exhibited a bimodal pattern, with peaks in the early morning and late evening, likely shaped by thermal constraints and prey movement. The more pronounced evening peak in winter may reflect avoidance of colder pre‐dawn hours by concentrating activity during the relatively warmer evening period, an adjustment also observed in other large carnivores under similar climatic constraints (Johansson et al. 2022; Ferreira and Viljoen 2022). Additionally, prey species such as ungulates reduce movement during the coldest hours before sunrise and become more active as temperatures increase, thereby influencing predator activity patterns (Marchand et al. 2014; Signer et al. 2011; Owen‐Smith 2008). In contrast, activity during the monsoon shifted to a unimodal pattern, characterised by elevated energy expenditure throughout the day and an evening peak. This shift may be shaped by changes in vegetation structure, reduced visibility, prey movement or variation in localised resource distribution (Dagtekin et al. 2024; Singh et al. 2024), which likely reduce the need for extensive movement. A similar temporal activity pattern was exhibited during the post‐dispersal phase, but dawn activity was lower in the monsoon than in winter, suggesting that seasonal conditions influence the intensity of dawn movement. Despite these temporal differences, the overall energy expenditure between monsoon and winter was similar, indicating that she maintained similar daily energetic budgets across seasons while varying diel activity. These patterns underscore the ability of the tigress to adjust its energetic strategies to environmental conditions.

Our study provides valuable insight into tiger fine‐scale activity and energy expenditure in a complex and human‐dominated landscape. However, the study is based on a single sub‐adult female, and therefore, the findings cannot be generalised to the broader tiger population or applied across sexes or landscapes. The absence of male data further restricts the interpretation of sex‐specific behavioural strategies, and the limited temporal and spatial scope may not capture broader ecological variability. Future studies with larger sample sizes encompassing multiple individuals, sexes and habitats are essential for drawing population‐level or landscape‐level conclusions. Nevertheless, this study provides a robust framework to apply high‐resolution tri‐axial accelerometry to quantify tiger energetics in the wild. These insights are valuable for informing conservation strategies in shared landscapes, where promoting coexistence requires a deeper understanding of large carnivore behaviour under human influence.

## Author Contributions

**Zehidul Hussain:** conceptualization (supporting), data curation (supporting), formal analysis (lead), methodology (equal), software (lead), writing – original draft (equal), writing – review and editing (equal). **Anjali Thapliyal:** data curation (supporting), formal analysis (supporting), writing – original draft (supporting), writing – review and editing (supporting). **Luca Börger:** methodology (equal), software (supporting), visualization (supporting), writing – review and editing (supporting). **Parag Nigam:** funding acquisition (supporting), investigation (supporting), methodology (supporting), writing – review and editing (supporting). **Bilal Habib:** conceptualization (equal), funding acquisition (equal), investigation (equal), methodology (equal), project administration (equal), supervision (equal), validation (equal), writing – original draft (equal), writing – review and editing (equal).

## Funding

Funding was provided by the Maharashtra Forest Department, Government of Maharashtra.

## Ethics Statement

Animals were captured following standard and approved protocols after due permission from the Ministry of Environment, Forests and Climate Change, Government of India and the Maharashtra Forest Department. The species was collared under the specific permit number: SPP‐04/2016.

## Consent

The authors have nothing to report.

## Conflicts of Interest

The authors declare no conflicts of interest.

## Data Availability

All data used in the analysis are uploaded as Supporting Information.
